# Emerging Roles of BRD7 in Pathophysiology

**DOI:** 10.3390/ijms21197127

**Published:** 2020-09-27

**Authors:** Sang Won Park, Junsik M. Lee

**Affiliations:** 1 Division of Endocrinology, Boston Children’s Hospital, Boston, MA 02115, USA; junsik.lee@childrens.harvard.edu; 2 Department of Pediatrics, Harvard Medical School, Boston, MA 02115, USA

**Keywords:** BRD7, glucose metabolism, type 2 diabetes, XBP1s, PI3K, p85, cancer

## Abstract

Bromodomain is a conserved structural module found in many chromatin-associated proteins. Bromodomain-containing protein 7 (BRD7) is a member of the bromodomain-containing protein family, and was discovered two decades ago as a protein that is downregulated in nasopharyngeal carcinoma. Since then, BRD7 has been implicated in a variety of cellular processes, including chromatin remodeling, transcriptional regulation, and cell cycle progression. Decreased BRD7 activity underlies the pathophysiological properties of various diseases in different organs. BRD7 plays an important role in the pathogenesis of many cancers and, more recently, its roles in the regulation of metabolism and obesity have also been highlighted. Here, we review the involvement of BRD7 in a variety of pathophysiological conditions, with a focus on glucose homeostasis, obesity, and cancer.

## 1. Introduction

Bromodomain is an evolutionarily conserved module, which consists of 110 amino acids [1,2]. It recognizes acetylated lysine residues through its hydrophobic cavity formed by four α-helices and two loops, and modulates the activity of other proteins [1,3,4,5]. The first protein reported to contain bromodomain was the Brahma protein, a regulator of *Drosophila* homeotic genes [6]. Since then, more than 60 different bromodomain sequences have been identified that can be categorized into 8 different families, based on structural similarities [7]. Bromodomain is found in a wide range of proteins and in most tissues, including metabolic, nervous, reproductive, and connective tissues [8,9]. Bromodomain-containing proteins serve critical roles in a variety of activities such as chromatin remodeling [10,11], transcriptional regulation [5], and cell signaling [9]. 

Bromodomain-containing protein 7 (BRD7) was first identified as a 75 kDa protein that interacts with the mouse protein tyrosine phosphatase BAS-like (PTP-BL) protein [12]. BRD7 is found in various tissues, including the lung, heart, muscle, kidney, brain, thymus, pancreas, skin, testes, liver, and colon [12], and is expressed at all stages of life, from fetus to adulthood [8,9]. Mice that lack BRD7 die by gestational day 16.5 [13], underscoring the protein’s crucial role during embryonic development. BRD7 binds to acetylated histones through its bromodomain and regulates transcription of cell cycle genes [14,15]. BRD7 is also a component of polybromo-associated BRG1-associated factor (PBAF), an ATP-dependent chromatin remodeling complex that is an analogue of the SWItch/Sucrose Non-Fermentable (SWI/SNF) complex [16]; it thus additionally modulates the transcription of genes as a regulator of SWI/SNF. Furthermore, BRD7 participates in the cell cycle, apoptosis, and cell progression (Table 1). Here, we review the role of BRD7 in a variety of clinical conditions (Table 2), with a focus on glucose metabolism and cancer. 

## 2. BRD7 in Metabolism

**Figure 1:**
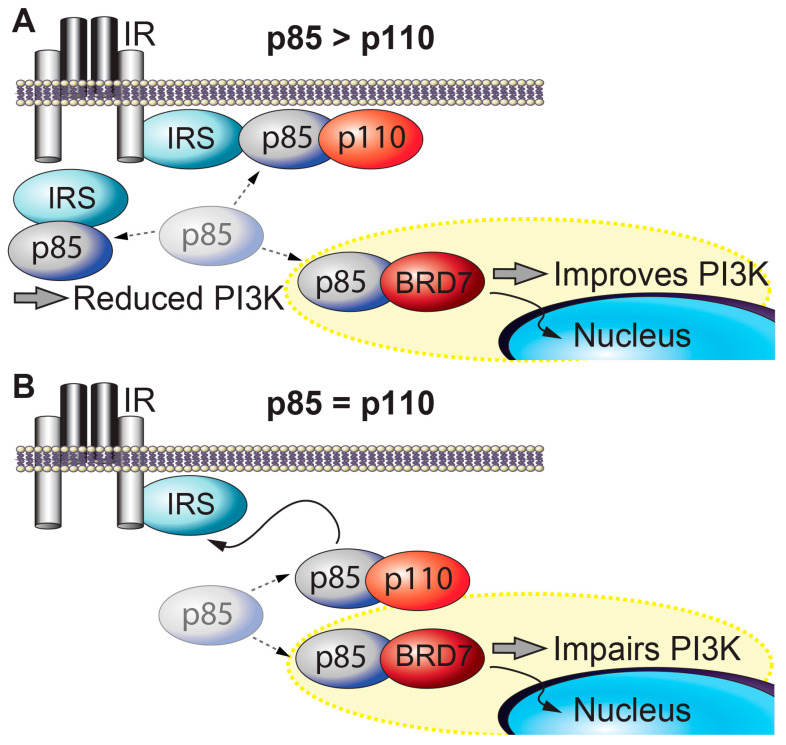
**BRD7 and insulin signaling.** (**A**) When p85 is present in excess of p110, free p85s inhibit PI3K (p85-p110) from binding to IRS. Overexpression of BRD7 removes free p85s from the cytoplasm to the nucleus and improves PI3K signaling. (**B**) When the amount of p85 and p110 is similar, BRD7 competes with p110 for binding to p85 and upregulation of BRD7 decreases PI3K signaling.

BRD7 and insulin signaling. Insulin activates insulin receptor, which recruits and phosphorylates its substrate adaptor proteins, such as the insulin receptor substrate (IRS) family proteins. Activated IRS proteins are recognized by the regulatory subunits of phosphatidylinositol 3-kinase (PI3K), p85α and p85β. p85 is required for the stability of the catalytic subunits of PI3K (p110α and p110β) and mediates the recruitment of p110 subunits to upstream activators. PI3K leads to activation of effector proteins, such as Akt. However, when p85 regulatory subunits are in excess of p110 catalytic subunits, p85s form homodimers that compete with p85-p110 heterodimers for binding to upstream activators of PI3K (e.g., IRS1) and impair insulin-induced PI3K signaling (Figure 1A) [47]. BRD7 regulates several major nodes in the insulin signaling pathway. BRD7 competes with p110 for binding to p85 [34]. BRD7 binds to the subset of p85 proteins that are not bound to p110, and transports these free p85 monomers to the nucleus, thereby increases insulin signaling (Figure 1A) [35]. However, in cells or tissues where the ratio of p85 to p110 is approximately 1:1, BRD7 upregulation decreases PI3K signaling by preventing p85 from binding to p110 (Figure 1B) [34]. Thus, the ratio of p85 to p110 serves a crucial role in cellular responses to insulin. Some tissues have approximately equal amounts of p85 and p110 proteins, while in other tissues (including the liver), p85 proteins are present in excess of p110 proteins. As a consequence, partial deletion of genes that encode various p85 isoforms improves insulin sensitivity [48,49]. Consistent with the biochemical model, knocking out both p85α and p85β in the liver leads to insulin resistance [50].

BRD7 increases phosphorylation of Akt at residues Thr308 and Ser473 and glycogen synthase kinase 3β (GSK3β) at residue Ser9, in the liver of mice as well as in mouse embryonic fibroblasts (MEFs) [36]. GSK3β is a target of Akt in the insulin signaling pathway and phosphorylation of GSK3β by Akt inactivates GSK3β, activates glycogen synthase (GS), and increases glycogen synthesis. Thus, BRD7 likely responds to insulin by inhibiting GSK3β through Akt [36]. Notably, BRD7 increases the phosphorylation of GSK3β even in the absence of Akt1/2 [36]. BRD7 also increases phosphorylation of S6K in the absence of Akt1/2 activity as well [36]. In response to insulin, activation of S6K occurs at a later time, when Akt phosphorylation levels start to decrease [51]. Therefore, it is possible that BRD7 immediately responds to insulin and affects phosphorylation of GSK3β through Akt, but maintains its activity on GSK3β through S6K, at a later time point when the immediate effects of insulin have abated. Overall, this would not only enhance glycogen synthesis following insulin stimulation, but also after Akt becomes inactive while nutrients are still abundant and glucose is still being utilized.

BRD7 in β cell function. Insulin and amylin are synthesized in pancreatic β cells and secreted in response to external stimuli. β cells comprise more than half of the cells in the pancreas [52], but the mass and function of these cells are decreased in diabetes [53]. BRD7 is a component of the polybromo-associated BRG1-associated factor (PBAF) chromatin remodeling complex [16], while BRD9, another member of the BRD family proteins, associates with the mammalian SWI/SNF (BAF) complex [54]. The PBAF-BRD7 and BAF-BRD9 complexes participate in the maintenance and survival of pancreatic β cells [24] through their interaction with the vitamin D receptor (VDR), which has been shown to contribute to the development of diabetes when defective [55]. BRD7 directs VDR to associate with PBAF, while BRD9 directs the association between VDR and BAF [24]. The VDR-PBAF-BRD7 linkage promotes a β cell stress response, while VDR-BAF-BRD9 suppresses the function of the VDR [24]. Treatment of INS1 cells with a combination of calcipotriol (a synthetic VDR ligand) and i-BRD9 (an inhibitor of BRD9) increases the interaction between VDR and PBAF-BRD7 [24]; this, in turn, results in coordinated transcriptional responses that suppress inflammatory responses while promoting β cell survival [24]. Treatment of *db*/*db* and streptozotocin-treated C57BL/6J mice with calcipotriol and i-BRD9 improves glucose tolerance, reduces fasting blood glucose levels, and increases insulin secretion [24]. In summary, BRD7 in the PBAF complex is involved in a VDR-dependent transcriptional program that is critical for β cell survival [24].

BRD7 and endoplasmic reticulum (ER) homeostasis. The ER is an organelle where secretory and membrane proteins are folded and modified into their functional structures. However, an accumulation of misfolded or unfolded proteins in the ER triggers a condition known as ER stress. Increased ER stress in turn activates a complex signaling cascade called the unfolded protein response (UPR) [56]. The three branches of the UPR consist of PKR-like ER kinase (PERK), activating transcription factor 6 (ATF6), and inositol-requiring enzyme 1 (IRE1). Activation of IRE1 by autophosphorylation enables the endoribonuclease activity of IRE1 and cleaves the mRNA of a transcription factor called X-box binding protein-1 (XBP1) [57,58], leading to translation of a higher molecular weight protein, namely the spliced form of XBP1 (XBP1s) [59,60,61,62]. XBP1s is a master regulator of ER function and protein folding capacity [63,64,65,66,67], and a lack of one allele of XBP1 leads to the development of obesity and dysregulation of glucose homeostasis [56]. Increased ER stress and activation of the UPR are observed in obesity [56] and lead to the development of insulin resistance [56].

**Figure 2:**
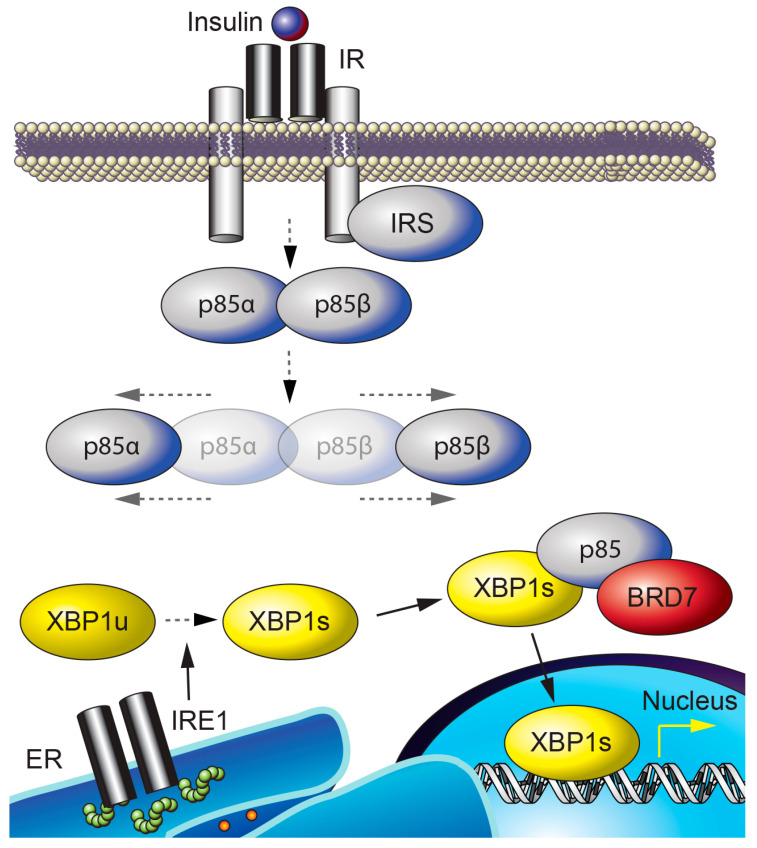
**BRD7 and ER homeostasis.** p85α and p85β form a heterodimer that is dissociated by insulin stimulation. IRE1 is activated by autophosphorylation upon ER stress and cleaves the unspliced form of XBP1 (XBP1u), resulting in XBP1s. BRD7, p85, and XBP1s interact to form a complex that is required for the nuclear translocation of XBP1s.

BRD7 interacts with p85 [34,35] through the iSH2 domain of p85 [34]; this interaction is required for p85-mediated nuclear translocation of XBP1s and for upregulation of XBP1s target genes that act to reduce ER stress (Figure 2) [35,68]. XBP1s is translocated to the nucleus in response to refeeding after a period of fasting, and relieves the ER stress caused by refeeding in the liver of lean wild-type mice [68]. In contrast, the nuclear translocation of XBP1s in response to refeeding is impaired in the liver of obese mice, and leads to increased ER stress [68]. Compared to lean mice, obese mice have significantly reduced hepatic BRD7 levels [35]. Use of an adenovirus-mediated transfer system to overexpress BRD7 in the liver of genetically obese *ob*/*ob* and high-fat diet-induced obese mice induces the nuclear translocation of XBP1s, resulting in increased transcription of XBP1s target genes and a decrease in ER stress levels [35]. Upregulation of BRD7 in the liver of obese and type 2 diabetic mice reduces blood glucose levels and improves glucose homeostasis [35,42]. These findings suggest that BRD7 participates in UPR signaling and promotes euglycemia by restoring the activity of XBP1s [35,69]. 

BRD7 and obesity. Obesity is a major risk factor for many life-threatening complications, including diabetes [70], cardiovascular diseases [71,72], and cancer [73]. The worldwide prevalence of obesity has nearly tripled over the last 30 years [74]. Obesity leads to insulin resistance and is closely associated with type 2 diabetes; the global incidence of type 2 diabetes has paralleled that of obesity such that the number of adults with type 2 diabetes has quadrupled in the last four decades [75]. However, the mechanistic links between obesity, insulin resistance, and the development of type 2 diabetes remain elusive.

Hepatic BRD7 levels are significantly reduced in genetically obese *ob*/*ob* and high-fat diet-induced obese mice [35]. Upregulation of BRD7 in the liver protects against the development of obesity. BRD7 transgenic mice, in which hepatic BRD7 levels are elevated, do not gain as much body weight as wild-type control mice do when they are placed on a high-fat diet (45% kcal from fat) [42]. BRD7 transgenic mice that are fed on a high-fat diet also display reduced plasma triglyceride levels [42] and decreased mRNA expression of *Pparγ*, *Fasn*, *Dgat2*, and *Srebf1* in white adipose tissue, showing that upregulation of BRD7 in the liver affects the expression of lipogenic genes in other organs [42]. These findings suggest that BRD7 is an important regulator of the development of obesity and lipid metabolism. 

Conversely, mice with a mixed 129/SvJ and C57BL/6J background that lack one allele of BRD7 display increased body weight when placed on a high-fat diet [42] and exhibit high levels of hepatic triglyceride [42]. Liver-specific BRD7 knockout mice (on the same background) that are fed on a high-fat diet also display increased body weight and elevated hepatic triglyceride levels [42]. These studies indicate that a lack of hepatic BRD7 leads to the development of obesity. On the other hand, neither heterozygous whole-body BRD7 knockout mice nor liver-specific BRD7 knockout mice exhibit significant disruptions in glucose homeostasis, even when they are fed a high-fat diet [42]. This may be because of the difference in hepatic BRD7 levels between BRD7 knockouts and control mice that are fed on a high-fat diet is not enough to show strong phenotypic changes [13], given that the high-fat diet significantly reduces hepatic BRD7 levels [42]. However, knocking down BRD7 by BRD7-specific shRNA in mice after challenging them with a high-fat diet leads to hyperglycemia [13]. 

## 3. BRD7 in Cancer

BRD7 was first discovered as a gene that is downregulated in nasopharyngeal carcinoma (NPC) biopsies [12]. Since then, decreased expression of BRD7 has been reported in many other human cancers including breast cancer [21], ovarian cancer [31], osteosarcoma [46], pancreatic cancer [76], colorectal cancer [41], and lung adenocarcinoma [77]. A bioinformatic study also suggests that the BRD7 gene is frequently deleted in a large set of human cancers [22]. A number of mechanisms have been proposed by which BRD7 expression is downregulated in cancer (Table 1 and Table 2): these include increased methylation of the BRD7 promoter [45,78], microRNA-mediated degradation of BRD7 mRNA [32,44,79], and increased ubiquitination of the BRD7 protein by E3 ligases [46,80]. Here, we summarize the role of BRD7 as a tumor suppressor in various organs. 

BRD7 in NPC. Since the initial discovery that levels of BRD7 are decreased in NPC [27], BRD7’s role as a tumor suppressor has been underscored in the HNE2 NPC cell line, in which BRD7 inhibits cell growth and G1-S cell cycle progression [27]. Upregulation of BRD7, in contrast, leads to differential expression of 12 genes that are involved in the MAPK/ERK and Rb/E2F signaling cascades [27]: these include the downregulation of MEK1, GRB2, and E2F3, and upregulation of CDC42. This suggests that BRD7 is involved in cell proliferation [28] and that the suppression of cell cycle progression by BRD7 occurs via the transcriptional regulation of key proteins involved in the cell cycle. Further studies in HNE1 NPC cells as well as COS7 cells show that BRD7 is localized primarily in the nucleus, and contains a nuclear localization signal (NLS) from amino acid residues 65 to 96 [81]. Mutant BRD7 that lacks the NLS remains primarily in the cytoplasm, and has a diminished ability to regulate the G1 to S progression as well as the expression of genes such as cyclin D1 and E2F3 [81].

Wnt/β-catenin signaling plays important roles in cell growth, proliferation, and survival, and overactivation of Wnt/β-catenin signaling is a common feature of cancer cells [82]. BRD7 inhibits the proliferation of HNE1 cells, by blocking the translocation of β-catenin to the nucleus. Negative regulation of β-catenin by overexpression of BRD7 downregulates the Wnt signaling pathway, as detected by decreased cyclin D1 and c-jun [30]. BRD7 also decreases phosphorylation of MEK and ERK, both of which are key proteins in the major pathways that promote cell cycle progression and cell proliferation [30]. On the other hand, BRD7 also reportedly enhances Wnt/β-catenin signaling. In HEK293T cells, BRD7 interacts with disheveled 1 (Dvl-1), a member of the intracellular scaffolding protein family; this interaction dephosphorylates GSK3β at residue Tyr216, and thereby inhibits its kinase activity [29]. By negatively regulating GSK3β, BRD7 enhances Wnt signaling, and also increases the nuclear translocation of β-catenin in COS-1 fibroblast-like cells [29]. How BRD7 differentially regulates β-catenin in different cell types and how this effect is related to its role as a tumor-suppressor remain to be resolved. Of note, overexpression of BRD7 negatively regulates β-catenin in cancer cells and positively regulates β-catenin in non-cancerous cells. 

**Figure 3:**
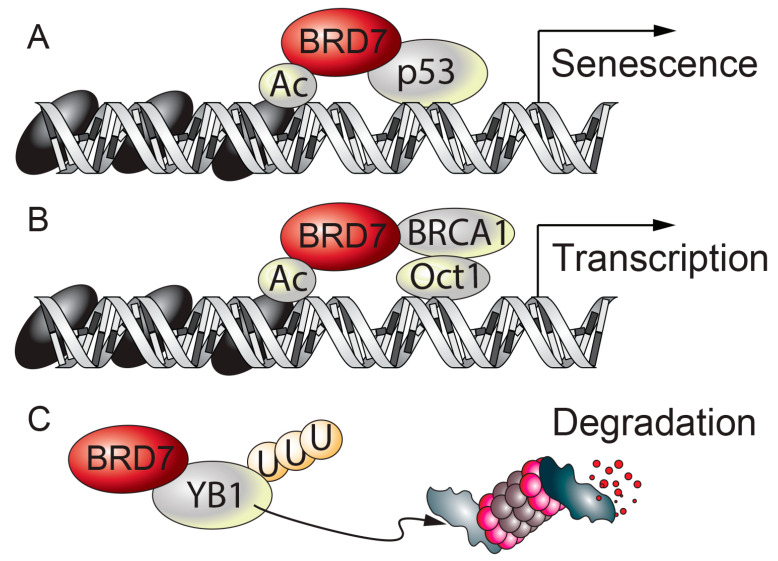
**BRD7 and cancer.** (**A**) BRD7 binds to p53 and increases the transcription of a subset of p53 target genes, leading to senescence. (**B**) BRD7 interacts with BRCA1 and recruits BRCA1 and Oct1 to the Esr1 promoter. (**C**) BRD7 binds to YB1 and leads to ubiquitin-mediated degradation of YB1. Ac: acetyl group. U: ubiquitin.

BRD7 in breast cancer. Although BRD7 was discovered in 1999, its role as a tumor suppressor was not well characterized until 2010, when several groups reported that BRD7 interacts with p53, a potent tumor suppressor that is frequently mutated in human cancers [83]. BRD7 binds to p53 through its N-terminus, where the NLS is located [21]; this interaction is required for p53 to transcriptionally activate a subset of p53 target genes, such as *P21*, *Hdm2*, *Ccng1*, *Rrm2b*, *Zmat3*, and *Fgf2* (Figure 3A) [21]. Yet other p53 target genes, such as *Bax, Fas, Tnfrsf10a, Tnfrsf10b,* and *Dram1*, do not require BRD7 as a co-transcriptional activator. Our understanding of how BRD7 selectively activates p53 is still incomplete. In the meantime, another group has reported that BRD7 is a p53-interacting partner, and also verified that only a subset of p53 target genes requires BRD7 as a co-activator of p53 [22]. Notably, depletion of BRD7 in BJ fibroblasts reduces expression of p21 (an inhibitor of cell cycle progression) in a p53-independent manner [22], and consequently delays proliferation and leads to premature senescence [84]. BRD7 also binds to p300, a histone-acetyl transferase that modifies histone tails, and the interaction between BRD7 and p53 is crucial for p300-dependent histone acetylation and also for p53 to upregulate gene transcription [21,85].

Around the same time as the above findings were published, BRD7 was identified as an interacting partner of BRCA1, another tumor suppressor associated with breast cancer, [23]; this interaction recruits BRCA1 and Oct1 to the Esr1 promoter, which encodes estrogen receptor α (ERα) and in turn increases acetylation of histone H3 (Figure 3B) [23]. Loss of BRD7 or BRCA1 in breast cancer cells prevents expression of ERα, and makes the cells resistant to fulvestrant [23], an antiestrogen drug that is often used to treat breast cancer. Moreover, through its N-terminus, BRD7 binds to Y box binding protein-1 (YB1) [40], an oncogene that is upregulated in certain cancers [86]; this in turn leads to ubiquitin-mediated degradation of YB1 in the MDA231 and MCF7 breast cancer cell lines (Figure 3C) [40]. Epithelial-to-mesenchymal transition (EMT), wherein epithelial cells lose their adhesive characteristics and acquire mesenchymal stem cell-like features, is a cellular process that is often induced in carcinoma. BRD7 inhibits the EMT process, and maintains the cells’ integrity in terms of the epithelial characteristics and low invasion ability [40].

BRD7 in female reproductive organs. Endometrial cancer, ovarian cancer, and cervical cancer are the most common reproductive cancers in women. Levels of BRD7 mRNA are decreased in serous ovarian cancer, especially in high-grade cancer [31]. Overexpression of BRD7 in A2780 and SKOV3 ovarian cancer cell lines reduces cell survival and increases apoptosis [31]. Furthermore, administration of BRD7 plasmids into mice that harbor an orthotopic A2780 cell line model of ovarian cancer leads to reduced tumor weight and decreased number of tumor nodules [31]. BRD7 negatively regulates β-catenin and thereby reduces MMP2 secretion in A2780 and SKOV3 cancerous cell lines [31]. Notably, BRD7’s effects on cell viability, apoptosis, and cell invasion occur independent of the presence of p53 in A2780 and SKOV3 cells [31]. In addition, the role of microRNA (miRNA)-200 family is indicated in several carcinomas [87,88]; in particular, miR-200c is upregulated in endometrial carcinoma [32]. Microarray analysis and RT-PCR results from the HEC-1A uterus adenocarcinoma cell line show that miRNA-200c decreases the expression levels of BRD7 [32]. Overexpression of BRD7 in the HeLa cervical cancer cell line attenuates PI3K activity, as shown by decreased Akt phosphorylation at the Ser473 residue [34].

BRD7 in other organs. Prostate cancer is one of the top three leading causes of death from cancer for men in the United States. Expression of BRD7 is reduced in patients with prostate cancer [89]. BRD7 binds to tripartite motif-containing 24 (TRIM24) [25], a member of the transcriptional intermediary family 1 (TIF1), which associates with chromatin and regulates gene expression [90]. The interaction between BRD7 and TRIM24 in prostate cancer cells negatively regulates TRIM24 activity, and diminishes cell proliferation and cell growth [25].

Osteosarcoma is a type of bone cancer that develops from malignant neoplasm of cells of mesenchymal origin, such as osteoblasts. In human osteosarcoma tissues, the anaphase-promoting complex/cyclosome (APC/C), a complex of E3 ligases that mediates the ubiquitination of proteins during mitosis [91,92], targets BRD7 for ubiquitin-mediated degradation through its co-activators cdh1 and cdc20 [46]. Increased interaction between BRD7 and cdh1 or cdc20 leads to ubiquitin-mediated degradation of BRD7 in osteosarcoma [46]. Inhibiting APC/C activity, which participates in promoting osteosarcoma cell growth and tumor formation, decreases degradation of BRD7 and reduces the proliferation of cells in osteosarcoma [46].

Non-small cell lung carcinomas (NSCLC) account for more than 80% of all lung cancers, and includes adenocarcinoma, squamous cell carcinoma, and large cell carcinoma. BRD7 expression is decreased in NSCLC [77], and low levels of BRD7 are associated with reduced survival rates in lung adenocarcinoma patients [77]. Also, BRD7 is a target of miR-410, an oncogene that is upregulated in NSCLC [44]. BRD7 also transcriptionally upregulates a tumor suppressor called X-linked inhibitor of apoptosis associated factor 1 (XAF1) in human lung microvascular endothelial cells (HMVECs) [26]. XAF1 upregulation in HMVECs reduces cell proliferation, and induces cell cycle arrest [26]. In lung adenocarcinoma cells, XAF1 expression induces cell cycle arrest, cell senescence, and apoptosis [91,92]. Given that BRD7 is required for XAF1 transcription [26], BRD7 likely serves a tumor-suppressive role in NSCLC. BRD7 also induces ferroptosis in hepatic stellate cells [93], which raises the possibility that BRD7 also plays a role as a tumor suppressor by regulating cell death through iron-dependent accumulation of lipid hydroperoxides [94]. Furthermore, BRD7 is downregulated in hepatocellular carcinoma [43], while higher BRD7 expression levels are associated with improved survival of patients with hepatocellular carcinoma, pointing to a tumor-suppressive role of BRD7 in hepatocellular carcinoma.

## 4. Discussion

Many studies on the role of BRD7 consist of observation-based research rather than in-depth mechanistic studies. Nonetheless, they have shown that BRD7 is involved in a diverse range of biological disciplines. Over the last two decades, BRD7’s diverse roles in multiple tissues and its involvement in a wide range of diseases have been highlighted, as delineated in this review. BRD7 was initially recognized as a tumor suppressor, but recent studies have provided evidence that BRD7 participates in the regulation of metabolism and insulin signaling pathway with detailed mechanistic underpinnings. 

Type 2 diabetes is characterized by hyperglycemia, insulin resistance, and glucose intolerance [95]. Of the therapeutic agents used to reduce symptoms of type 2 diabetes, metformin is the first-line treatment and works by reducing hepatic glucose production [96]. Thiazolidinediones act by increasing insulin sensitivity, while sulfonylureas and meglitinides increase insulin secretion [97]. Other medications are known to inhibit specific proteins involved in glucose metabolism, such as DPP-4, GLP-1 receptor, and SGLT2 [97]. However, none of these drugs can cure type 2 diabetes, and one of the largest obstacles to developing a cure is our incomplete understanding of how insulin resistance and glucose intolerance develop. One of the proposed underlying causes for the development of obesity and type 2 diabetes is ER stress [98]. XBP1s plays a vital role in metabolic regulation [56,98,99] and XBP1 haplodeficiency leads to the development of obesity and type 2 diabetic features [56]. The mechanism by which XBP1s is translocated to the nucleus to serve as an active transcription factor was discovered in 2010, when the interaction between XBP1s and p85s, a component of the insulin signaling pathway, was unveiled [68,100]. Although the p85-p110 PI3K complex participates in the insulin signaling pathway in the cytosol, some reports showed the presence of p85 in the nucleus [34,35,101,102,103]. In 2014, the interaction between p85s and BRD7 was found to be critical for the nuclear translocation of p85 [34,35] as well as of XBP1s [35]. These results collectively support the role of BRD7 in the regulation of ER and glucose homeostases. The involvement of BRD7 in the regulation of glucose homeostasis was shown in two major metabolic tissues: the liver [35] and the pancreas [24]. 

Two studies reported that BRD7 affects the activity of Akt, downstream of PI3K signaling. Overexpression of BRD7 can increase [35] or decrease [34] the phosphorylation of Akt in response to insulin, depending on the amount of p85 present in relation to p110. When the ratio of p85/p110 is high, excess p85 suppresses insulin signaling [47,104,105,106,107]. Under these conditions, reducing cytosolic p85 by BRD7-mediated nuclear translocation likely balances the ratio of p85 to p110, and enhances PI3K activity as well as Akt phosphorylation [35]. On the other hand, when p85 is not in excess of p110, PI3K is highly activated and leads to hyperactive Akt activity and increased cell proliferation [108]. Under these conditions, translocating p85 into the nucleus by BRD7 reduces PI3K activity and decreases phosphorylation of Akt [34]. The dynamics of p85 are complex, and it seems that the response of p85 varies depending on the cell environment. For example, p85 not only binds to p110 to activate the PI3K pathway, but also associates with PTEN, which in turn dephosphorylates PIP_3_ [109,110]. Likewise, upregulation of BRD7 improves insulin signaling in obesity, but inhibits PI3K activity in a cancerous state. Further studies are needed to unravel the complex roles of BRD7 and p85. However, given that the region of BRD7 that binds to p85 is highly conserved [34], it is likely that the role of BRD7 in PI3K signaling is related to more fundamental functions of p85 in the insulin signaling pathway.

Inhibition of GSK3β improves glucose tolerance in type 2 diabetic mice, while increased activation of GSK3β contributes to type 2 diabetes [111,112,113]. Therefore, GSK3β has been suggested as a therapeutic target for type 2 diabetes [113]. BRD7 enhances phosphorylation of GSK3β as well as S6K, and 4E-BP1, downstream effector proteins of mTORC1 [36]. Of interest, BRD7 increases phosphorylation of GSK3β and S6K even in the absence of Akt activity, but 4E-BP1 phosphorylation is not affected by BRD7 overexpression without Akt [36]. These observations suggest that BRD7 not only responds to insulin stimulation (via Akt), but it can also sense nutrient abundance such that GSK3β is kept inactive when there is a sufficient energy source after meals (via S6K) without affecting the global translation of proteins through the activity of 4E-BP1. How BRD7 is able to respond to insulin and nutrient availability, and the precise pathway involved in such regulation have not been elucidated yet. Meanwhile, an abundance of amino acids is important for cell maintenance, cell survival, and biosynthesis. In particular, glutamine is used as a substrate for many biosynthetic pathways and glutamine metabolism has been discussed in cancer cells [114,115,116]. Considering the general role of BRD7 as a tumor suppressor, its regulation of the G1-S transition in NPC cells, and involvement in nutrient-sensing pathways, it is possible that BRD7 participates in the late G1 metabolic checkpoints mediated by essential amino acids, glutamine, and mTOR [117]. After unraveling the mechanism of action, the next step is to find compounds that upregulate BRD7 activity for the purpose of treating pathophysiological conditions.

The fact that mice with whole-body BRD7 knockout fail to survive embryonic development clearly points to a critical role for BRD7 in the normal functioning of cells and tissues [13]. In this mouse model, a cassette with β-galactosidase and the neomycin-resistance gene induces a frameshift and renders the BRD7 gene non-functional [13]. Of note, some studies used BRD7 whole-body knockout mice, which were generated with use of the Cre/LoxP recombination system, expressing Cre under the EIIα promoter [118,119]. Cre/LoxP is a useful technology that has been widely used for tissue-specific genetic manipulation. However, under certain conditions, Cre mosaicism can lead to incomplete recombination in all cells, which then can exhibit inconsistent activity [120]. In particular, it was shown that commercially available EIIα-Cre mice can display mosaic recombination depending on whether the gene was inherited paternally or maternally [120]. Therefore, the possibility of having an incomplete recombination in the target tissues should be carefully evaluated. Regardless, the Cre/LoxP recombination system is generally accepted as a powerful tool for manipulating the genotypes of animals and has produced many significant discoveries. In light of this, we speculate that BRD7 knockout mice generated by the Cre/EIIα promoter may survive even with a low expression level of BRD7 that is undetectable by standard methods for verifying genetic knockout. Nonetheless, they display strong phenotypes, such as male infertility [118] or impaired cognitive behavior [119].

## 5. Conclusions

Downregulation of BRD7 has been shown to be associated with many pathophysiological conditions, including obesity, diabetes, and cancer. Many reports also emphasize that upregulation of BRD7 alleviates or prevents the development of these pathological conditions. Additional investigations are needed to further uncover BRD7’s mechanism of action. Nonetheless, the body of published data so far suggest that BRD7 can be an attractive therapeutic target for treating patients with type 2 diabetes and cancer. 

## Figures and Tables

**Table 1:** **BRD7’s function.** The role of BRD7 is summarized by cellular function.

Function	Mechanism	Ref
**Chromatin Remodeling**	BRD7 binds to acetylated histone H3 through its bromodomain and BRD7 is involved in chromatin remodeling.	[14]
	The bromodomain of BRD7 contains the left-handed four-helix bundle topology, which binds to acetylated lysine on histone H3 or H4.	[17]
	BRD7 is a component of the PBAF chromatin remodeling complex.	[16]
**Transcriptional Regulation**	BRD7 interacts with interferon regulatory factor 2 (IRF2).	[18]
	Seh1 recruits Olig2 and BRD7 to form a complex and increases the transcription of myelination-associated genes and chromatin modification.	[19]
	BRD7 interacts with E1B-AP5, which is involved in mRNA processing and transport. The complex associates with histones.	[15]
	BRD7 binds to Smad proteins and increases TGFβ-Smad-dependent transcriptional activity.	[20]
	BRD7 interacts with p53 and regulates the transcriptional activity of a subset of p53 target genes involved in induction of replicative and oncogenic stress senescence.	[21,22]
	BRD7 facilitates the recruitment of BRCA1 and Oct-1 to the ESR1 promoter and regulates the transcription of ERα.	[23]
	The association between vitamin D receptor and PBAF is increased by BRD7 in β cells, leading to transcriptional activation of genes involved in anti-inflammatory responses and maintenance of β cell function.	[24]
	BRD7 negatively regulates the transcriptional activity of androgen receptor in the CWR22Rv1 prostate cancer cell line by binding to TRIM24, an activator of androgen receptor.	[25]
	BRD7 is required for the expression of the tumor suppressor XIAP-associating factor 1 in human pulmonary microvascular endothelial cells.	[26]
**Cell Cycle Progression**	Overexpression of BRD7 in HNE2 cells inhibits the G1-S phase transition and downregulates expression of proteins in the ras/MEK/ER and E2F/Rb pathways.	[27]
	BRD7 attenuates ras/raf/MEK/ERK signaling and represses cell proliferation.	[28]
**Wnt Signaling**	BRD7 interacts with DVL1 and promotes Wnt signaling in HEK293T cells in a DVL1-dependent manner by inhibiting the activity of GSK3β and increasing the nuclear translocation of β-catenin.	[29]
	BRD7 overexpression in HNE1 nasopharyngeal carcinoma cells inhibits nuclear accumulation of β-catenin.	[30]
	BRD7 negatively regulates the β-catenin pathway in A2780 and SKOV3 ovarian cancer cell lines by inhibiting nuclear translocation of β-catenin.	[31]
	BRD7 expression is inhibited by microRNA-200c in HEC-1A and Ishikawa endometrial carcinoma cells, which leads to increased nuclear translocation of β-catenin and consequent increased transcription of cyclin D1 and c-myc.	[32]
	BRD7 transcriptionally upregulates miR-3148 in C33A cells, which reduces Wnt3a expression, and thus inhibits oncogenic Wnt3a/β-catenin signaling.	[33]
**Insulin Signaling**	BRD7 interacts with p85α/β, the regulatory subunits of PI3K, and increases their nuclear translocation. This increases PI3K-Akt signaling in the liver, but attenuates Akt activity in HeLa cervical cancer cells.	[34,35]
	BRD7 increases phosphorylation of glycogen synthase kinase 3β (GSK3β) at residue Ser9, which leads to inactivation of GSK3β.	[36]
**Unfolded Protein Response**	BRD7 increases the nuclear translocation of the spliced form of X-box binding protein 1 (XBP1s), upregulates the transcription of XBP1-target genes, and relieves ER stress.	[35]
	BRD7 is required for hyperglycemia-induced apoptosis in H9c2 cardiomyoblasts. Reduction of BRD7 in the heart of diabetic rats alleviates ER stress-induced myocardial apoptosis.	[37]
**Inflammation**	MEFs that lack BRD7 and EIIα-Cre-derived BRD7-deficient mice show increased nuclear translocation of p65 and NF-κB transcriptional activity.	[38]
	BRD7 knockdown in ApoE-knockout mice promotes atherosclerotic lesion formation and vascular inflammation by promoting the transcriptional activity of NF-κB.	[39]

**Table 2:** **BRD7 and pathophysiology in various tissues.** The role of BRD7 in diseases of various tissues is listed.

Tissue Type	Disease	Mechanism of Progression	Ref.
**Breast**	Cancer	The BRD7 locus is frequently found deleted in human breast tumors. BRD7 is required for p53-mediated transcription of a subset of p53 target genes.	[21]
	Cancer	BRD7 overexpression suppresses the epithelial-mesenchymal transition and metastasis of breast cancer cells through increasing degradation of the oncogenic protein YB1.	[40]
**Colon**	Cancer	BRD7 is downregulated in colorectal cancer tissues. BRD7 expression level is correlated with survival time in colorectal cancer patients.	[41]
**Liver**	Obesity	BRD7 levels are decreased in the liver of genetically obese *ob*/*ob* and high-fat diet-induced obese mice.	[35]
	Obesity	Heterozygous whole-body and liver-specific knockout of BRD7 leads to increased weight gain in mice, exacerbated by high-fat diet feeding. Long-term upregulation of hepatic BRD7 reduces weight gain in mice challenged with a high-fat diet.	[42]
	Type 2 diabetes	Upregulation of BRD7 in the liver of obese and type 2 diabetic mice decreases blood glucose levels and improves glucose homeostasis.	[35,42]
	Cancer	BRD7 is downregulated in hepatocellular carcinoma (HCC), and higher BRD7 levels are correlated with improved outcomes in HCC patients. BRD7 inhibits HCC tumor growth in a xenograft mouse model.	[43]
**Lung**	Cancer	BRD7 expression levels are downregulated in non-small cell lung cancer (NSCLC). Upregulated expression of microRNA-410 in NSCLC leads to decreased BRD7 expression and increased Akt phosphorylation.	[44]
**Nasopharynx**	Cancer	BRD7 expression levels are downregulated in nasopharyngeal carcinoma.	[27]
	Cancer	High methylation frequency of the BRD7 promoter is found in tumor and blood samples of patients with nasopharyngeal carcinoma.	[45]
**Bone**	Cancer	BRD7 is degraded by anaphase promoting complex in U2OS osteosarcoma cells. Upregulation of degradation-resistant BRD7 reduces cell growth and tumorigenesis.	[46]
**Ovary**	Cancer	The expression of BRD7 is decreased in high-grade serous ovarian cancer tissues. Overexpression of BRD7 in A2780 and SKOV3 ovarian cancer cell lines increases apoptosis and inhibits cell migration.	[31]
**Pancreas**	Diabetes	BRD7 increases the association between vitamin D receptor and PBAF in β cells. This complex maintains β cell function and reduces glucose levels in *db*/*db* and streptozotocin-treated mice.	[24]

## Abbreviations

BRD7	Bromodomain-containing protein 7
PTP-BL	Protein tyrosine phosphatase BAS-like protein
PBAF	Polybromo-associated BRG1-associated factor
SWI/SNF	SWItch/Sucrose Non-Fermentable complex
PI3K	Phosphatidylinositol 3-kinase
GSK3β	Glycogen synthase kinase 3β
MEF	Mouse embryonic fibroblast
GS	Glycogen synthase
ER	Endoplasmic reticulum
UPR	Unfolded protein response
PERK	PKR-like ER kinase
ATF6	Activating transcription factor 6
IRE1	Inositol-requiring enzyme 1
XBP1	X-Box Binding Protein-1
XBP1s	The spliced form of XBP1
NPC	Nasopharyngeal carcinoma
NLS	Nuclear localization signal
Dvl-1	Disheveled 1
ERα	Estrogen receptor α
TRIM24	Tripartite motif-containing 24
TIF1	Transcriptional intermediary family 1
APC/C	Anaphase-promoting complex/cyclosome
NSCLC	Non-small cell lung carcinomas
XAF1	X-linked inhibitor of apoptosis associated factor 1
HMVEC	Human lung microvascular endothelial cell
